# Percentages of Cases in Operating Rooms of Sufficient Duration to Accommodate a 30-Minute Breast Milk Pumping Session by Anesthesia Residents or Nurse Anesthetists

**DOI:** 10.7759/cureus.12519

**Published:** 2021-01-06

**Authors:** Sarah Titler, Franklin Dexter, Richard H Epstein

**Affiliations:** 1 Anesthesiology, University of Iowa, Iowa City, USA; 2 Anesthesiology, University of Miami Miller School of Medicine, Miami, USA

**Keywords:** lactation, academic medical centers/organization and administration, postnatal care, breastfeeding

## Abstract

Introduction: Accommodating breast milk pumping sessions is required by US federal statute, but fulfillment is challenging for US anesthesia providers (e.g., anesthesia residents and nurse anesthetists). Considerations of good anesthesia practices (e.g., being present for critical portions of cases, including induction and emergence) create limits on which procedures are suitable for such relief. Our objective was to quantify the minimum percentages of cases for which there could reliably (≥ 95%) be at least 30 minutes during the surgical time when the anesthesia provider could receive such breaks.

Methods: We studied all surgical cases performed at an anesthesia department over four years, including its inpatient surgical suite, pediatric hospital, and ambulatory surgery center. The 5% lower prediction bounds of surgical times (surgery or procedure start to end) were calculated from three years of historical data (October 1, 2016, to September 30, 2019) based on two-parameter lognormal distributions. The prediction bounds were compared to actual surgical start times during the next one year (October 1, 2019, to September 30, 2020). We considered the interval available for a breast milk pumping session during a case to be from 15 minutes after the start of the surgical time (to allow completion of initial documentation, other activities, and hand-off to the relieving anesthesia provider) until the end of the surgical time.

Results: The lower prediction bounds were accurate, with 4.9% (4.6% - 5.2%) of future cases’ surgical times being briefer, matching the nominal 5.0% rate. Applying these bounds, approximately 39% of cases (99% confidence interval 39% - 40%) were reliably of sufficient duration for the anesthesia provider delivering care in that one operating room to receive a 30-minute break for breast milk pumping session between 15 minutes after the start of surgery and procedure end. This percentage (39%) was substantially less than the 72% of the surgical times that were observed, in retrospect, to be sufficiently long because the lower 5% prediction bounds accounted correctly for the uncertainty in the duration of each case. The observed 39% prevalence was significantly fewer than half the cases (P < 0.0001 vs. 50%) suitable for such relief.

Conclusions: Individuals making operating room assignments for anesthesia providers need to consider the 5% lower prediction bounds of surgical times for cases in the room when making such assignments for women who require time for breast milk pumping sessions. Such considerations will generally result in assignments to rooms with one or more long-duration cases. Such a strategy may involve changes in preferred assignments for the anesthesia providers and alteration in the order of rotations for anesthesia residents (e.g., palliative care rotation rather than transition to practice at a pediatric ambulatory surgery center). When making room assignments for anesthesia providers who are breastfeeding, our results show that the 5% lower prediction bounds of surgical times need to be calculated; relying on the average surgical times for procedures is insufficient. Our paper also shows how to perform the mathematics using a spreadsheet program or equivalent.

## Introduction

The American Academy of Pediatrics recommends that infants be fed only breast milk for the first six months of life and continue to receive breast milk for at least the first year of life (e.g., to reduce infants’ gastrointestinal infections). Breastfeeding also has maternal benefits [1]. The US Fair Labor Standards Act requires “reasonable break time” for a woman “to express breast milk for her nursing child for 1 year after the child’s birth each time such employee has need to express the milk” [2]. The Accreditation Council for Graduate Medical Education (ACGME) includes “clean and private facilities for lactation that have refrigeration capabilities, with proximity appropriate for safe patient care” and "the time required for lactation" as common program requirements for accredited residency and fellowship programs [3].

Among US women anesthesiologists who had pregnancies resulting in childbirth during their residency or fellowship training, breaks for lactation sessions increased from 69% (159/229) of pregnancies between 2000 and 2010, to 83% (252/305) of pregnancies between 2011 and 2018, and to 97% (32/33) among current trainees (P < 0.0001). There were also more months of breastfeeding, mean (standard deviation) 7.7 (7.1) months [median 6], 9.6 (5.7) months [median 10], and 9.3 (6.6) months [median 9], respectively (P < 0.0001) [4]. However, surveyed anesthesiologists in the USA reported a lack of adequate facilities or insufficient time to express breast milk for 55% (57/104) of pregnancies [5]. Also, surveyed anesthesia program directors in New Zealand reported significantly less implementation than the perceived importance of “family-friendly” workplace practices, including designated time for breast milk pumping [6].

Being relieved during an ongoing case for a breast milk pumping session means, ideally, that anesthesia induction is complete, patient positioning is complete, and initial documentation is done. These steps are complete in 50% of cases 12 to 13 minutes after surgical incision, depending on the surgical procedure. Neither surgical time, neuraxial anesthesia, positioning, ambulatory surgery center, or specialty influenced the 12- to 13-minute duration [7]. Several extra minutes also are needed for the handoff to the relieving provider and for the trip to the lactation room, which may not be close to the surgical suite. Therefore, in the current study, we examined the percentage of surgical cases that reliably (≥ 95%) had at least 30 minutes during the interval from 15 minutes after surgical incision, or its equivalent, until surgery or the procedure has been completed (e.g., surgical dressing applied) [8,9]. The latter endpoint allows the provider receiving the break to return in time for tracheal extubation or supraglottic airway removal, whether the individual is a nurse anesthetist, anesthesiologist assistant, anesthesia resident, or anesthesiologist personally performing the anesthetic. Timing the breast milk pumping session so that the provider returns by the end of surgery is important because communication and report at the end of the > 30 minutes away generally will not occur during the series of critical events at the end of the case (e.g., turning the patient from prone back to supine, tracheal extubation). If the provider returns from a break after the end of surgery, when attention needs to be focused on the patient, the handoff may take considerably longer than if it occurred during a less work-intensive phase of the case. This may result in a delay in providing breaks in other rooms. For the anesthesia residents, there also would be reduced educational benefit from being absent for the end of cases. We previously showed that when the anesthesia provider had finished fewer than five previous cases with a neurosurgeon performing glioma surgery then there was greater incidence of prolonged (≥ 15 minutes) time to tracheal extubation [10].

We hypothesized that, overall, most cases (> 50%) would not have a period reliably of sufficient duration to accommodate a breast milk pumping session for the anesthesia provider. We also expected that this percentage would be heterogeneous among sites, significantly greater at an ambulatory surgery centers with its many brief duration cases, and significantly less at an inpatient, adult surgical suite with more long-duration cases. If true, the implication of the finding of overall > 50% of cases not suitable for relief would be that the individual(s) responsible for making operating room (OR) assignments for anesthesia providers who are breastfeeding need to consider the lower prediction intervals of cases in the room in which the provider will be working that day.

## Materials and methods

The University of Iowa Institutional Review Board determined that this project does not meet the regulatory definition of human subjects research because the activity is limited to the analysis of deidentified data provided for routine administrative purposes.

Data analyzed

We studied four years of data from a large teaching hospital. Substantively long historical periods for estimating surgical times can cause bias in estimates because of changes over time in procedures, positioning, etc. Brief periods result in smaller sample sizes and less precise estimates. In a previous study of estimating Bayesian parameters for lower and upper prediction bounds at three hospitals (University of Iowa, Thomas Jefferson, and Vanderbilt), three years of historical data were successfully used to balance these objectives [11]. We used the same duration of three years of data for the historical period: October 1, 2016, through September 30, 2019. Comparison was made with the following year: October 1, 2019, through September 30, 2020, the contemporaneous period. We conducted the analysis by year to avoid potential issues related to seasonal variation in the relative distribution of different surgical procedures.

Demographic data of the historical and contemporaneous periods are shown in Table 1 [12]. The table is organized by counts of cases, not OR days. The reason is that if a mother breastfeeds or pumps before leaving for work, then she may need a breast milk pumping session during a case in the middle to late morning, not deferred until later in the workday. Thus, our unit of analysis was the case, not the workday. Table 1 includes the distributions of patients who are American Society of Anesthesiologists Physical Status 1 or 2, OR times, and whether cases are elective. These are provided because the probability distributions of these factors vary significantly among ORs (i.e., they relate to anesthesia assignments). For example, a freestanding surgery center both has a greater percentage of cases among patients’ physical status 1 or 2 and briefer surgical times than does an inpatient surgical suite [13].

**Table 1:** Demographics, Surgical Durations, and Anesthesia Times of the Cases at the Different Surgical Suites and Periods Abbreviations: ASA, American Society of Anesthesiologists; min, minutes

	Historical Period	All Cases Combined	Ambulatory Surgery Center	Children's Hospital	Children's Hospital Elective Cases	Adult Surgical Suite	Adult Surgical Suite Elective Cases
Cases	95,146	30,987	8121	5004	4254	17,862	13,665
Surgical times (min)							
5^th^ percentile	12	12	10	8	8	21	22
25^th^ percentile	38	40	24	28	28	61	68
50^th^ percentile	80	82	51	55	55	110	119
75^th^ percentile	143	146	89	107	109	182	193
95^th^ percentile	302	302	174	246	251	350	370
% $\geq$ 45 min	71.0%	72.2%	55.8%	58.6%	58.6%	83.5%	85.7%
Anesthesia times (min)							
5^th^ percentile	18	19	13	12	11	31	32
25^th^ percentile	37	37	25	34	33	49	50
50^th^ percentile	53	53	35	48	46	63	63
75^th^ percentile	71	71	46	67	65	81	80
95^th^ percentile	112	109	63	115	115	117	114
ASA Physical status							
Physical status 1 or 2	59.8%	59.0%	78.2%	73.9%	75.2%	46.0%	51.4%
Median surgical times (min)	72	74	54	50	51	110	116
95^th^ percentile surgical times (min)	265	265	178	211	217	315	327
Surgical Specialties (%)							
Orthopedics	24.5%	26.6%	43.5%	16.4%	14.3%	21.8%	23.1%
General surgery	22.5%	21.8%	10.3%	20.8%	18.2%	27.4%	25.7%
Urology and gynecology	12.5%	13.7%	9.1%	15.7%	17.3%	15.2%	17.4%
Otolaryngology and dentistry	14.1%	12.7%	4.2%	30.7%	34.3%	11.6%	13.1%
Ophthalmology	11.3%	10.2%	30.7%	8.2%	9.2%	1.5%	1.1%
Neurosurgery	9.3%	9.5%	1.8%	5.0%	3.5%	14.2%	11.9%
Cardiothoracic surgery	3.8%	3.6%	0.0%	3.0%	3.2%	5.4%	5.7%

During the contemporaneous period (October 1, 2019, to September 30, 2020), there was a lactation room with a refrigerator adjacent to the surgical suite of the ambulatory surgery center and one adjacent to the surgical suite of the children’s hospital. The two anesthesia call rooms near the adult surgical suite were reserved as lactation rooms from 8:00 to 17:00 during regular workdays; both rooms were equipped with refrigerators.

The OR time (i.e., “wheels in” to “wheels out”) was divided into two portions, the surgical time and the anesthesia time. We considered the surgical time to be the period from surgery begin (the documented incision or, if not relevant, the procedure start time) to surgery end (application of the surgical dressing or, if not relevant, the procedure end time). We considered the anesthesia time to equal the OR time minus the surgical time. Our anesthesia time is longer than the anesthesia-controlled time, as the latter excludes the period from the start of positioning and surgical draping until incision. We did that because our prior study showed that documentation and other events were completed in 50% of cases by 12 to 13 minutes after the start of the surgical procedure [7].

Calculation of prediction bounds

**Figure 1:**
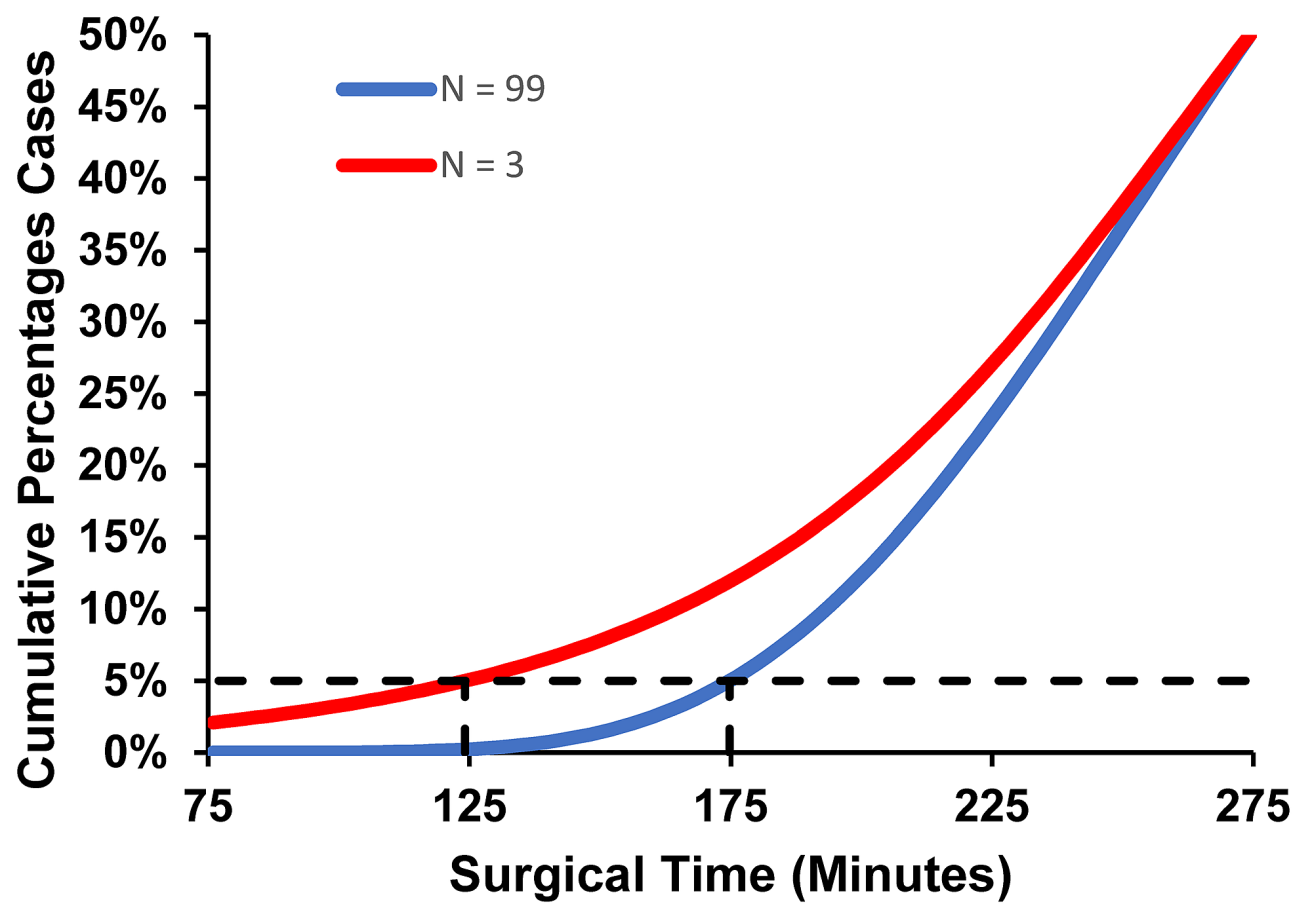
Cumulative Distribution Functions for Surgical Times Cumulative distribution functions for surgical times of Healthcare Common Procedure Coding System 55866, “Laparoscopy, surgical prostatectomy, retropubic radical, including nerve sparing, includes robotic assistance.” The blue line shows the left side of the fitted lognormal distribution curve from Figure 2. The red line shows the exponential of the Student t-distribution curve based on N = 3 cases, using the same parameter estimates (mean and standard deviation in the log scale) as for the blue line. Among the 2596 procedures during the 1-year contemporaneous period, there were N ≤ 3 cases from the 3-year historical period for 26% (673/2596) of the procedures. Both lines have the same median of 274 minutes (i.e., the lines intersect at the cumulative percentage of 50%), because the median in the arithmetic time scale is the exponential of the mean in the log scale. Whereas the 5th percentile of the lognormal distribution is 176 minutes, the 5% lower prediction bound calculated using the Student t-distribution is 175 minutes for N = 99 historical data versus 124 minutes for N = 3 historical data. The 5% lower prediction bound with only N = 3 historical cases is much less because there is considerable uncertainty in the estimated mean and standard deviation.

The figures were created using the current paper’s data, matching the corresponding figures in our 2000 paper on lower prediction bounds [14]. Figure 1 shows how prediction bounds depend on sample sizes.

**Figure 2:**
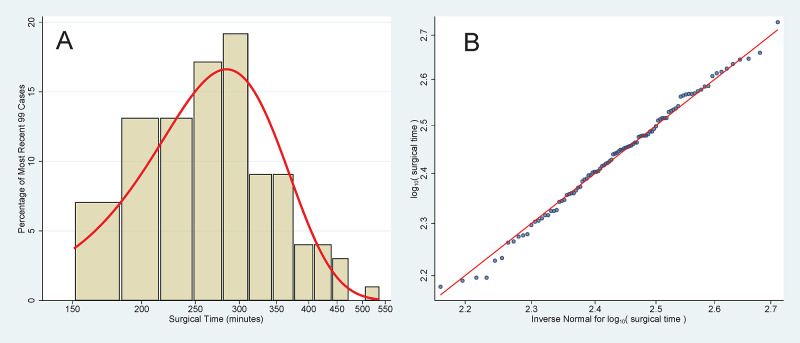
Distribution of Surgical Times Surgical times of the most recent 99 cases comprising the historical period for Healthcare Common Procedure Coding System 55866, “Laparoscopy, surgical prostatectomy, retropubic radical, including nerve sparing, includes robotic assistance.” From the 3-year historical period, there were 3478 other procedures performed among the 95,146 cases. The Shapiro-Wilk test applied to the logarithm of duration shows an excellent fit to a normal distribution, P = 0.84. (A) Histogram in log scale, along with a superimposed normal density plot. (B) Normal quantile plot, along with the reference line.

Figure 2 demonstrates an excellent fit to a lognormal distribution for the same example procedure as used in Figure 1. A prediction bound for a single future observation is a value that will, with a specified degree of confidence, be exceeded by the next randomly selected observation from a population. The specified degree of confidence then equals one minus the prediction bound [14]. Thus, a 5% prediction bound provides a 5.0% risk of the surgical time being too brief for a breast milk pumping session when an anesthesia resident or nurse anesthetist is given a break and will return before waking the patient up from general anesthesia. Equivalently, there would be a 95% degree of confidence that the pumping session could be accommodated. For small numbers of historical cases, the value for the 5.0% prediction bound can be substantially less than the value of the 5th percentile [14], as demonstrated in Figure 1.

Overall, 72.2% of the cases during our contemporaneous period had surgical times $\geq$ 45 minutes (Table 1). In contrast, 39.3% of the cases were sufficiently long, and with sufficiently many historical cases, that each future case individually had a reliable ($\geq$ 95%) chance of lasting at least 45 minutes (Table 2).

**Table 2:** Characteristics of 5% Lower Prediction Bounds of Surgical Times Abbreviations: min, minute

	All Cases Combined	Ambulatory Surgery Center	Children's Hospital	Children's Hospital Elective Cases	Adult Surgical Suite	Adult Surgical Suite Elective Cases
5% lower prediction bounds estimated with lognormal	30,357	7987	4872	4163	17,498	13,411
% surgical times excluded, < 2 historical period cases Denominator is row 1 of Table 1, Count of cases	2.0%	1.7%	2.6%	2.1%	2.0%	1.9%
5% lower prediction bound results						
% underestimated, denominator is row 1	4.86%	5.15%	5.19%	4.90%	4.56%	4.61%
lower 99% confidence interval	4.55%	4.37%	4.34%	4.49%	4.10%	4.02%
upper 99% confidence interval	5.19%	6.02%	6.14%	5.33%	5.04%	5.25%
5% lower prediction bounds $\geq$ 45 min						
%, denominator is row 1	39.3%	18.0%	20.7%	21.2%	54.2%	59.7%
lower 99% confidence limit	38.6%	16.9%	19.2%	19.6%	53.2%	58.6%
upper 99% confidence limit	40.0%	19.1%	22.2%	22.9%	55.2%	60.8%

A prediction bound incorporates two sources of variability in the estimate of surgical time. First, there is variability intrinsic to the scheduled procedure, as shown in Figure 2. This source of variability exists whether there are two previous cases’ durations or hundreds of previous cases’ durations. Second, there is variability in the parameter estimates for the distribution of times. For procedures with few previous cases’ durations available to estimate parameters, the estimated parameter values may differ from what would have been estimated with many (e.g., 99) previous cases’ surgical times [14]. The lack of historical data and resulting parameter uncertainty accounts for a substantial proportion of the variability (e.g., 21.1% [standard error 1.2%] of the resulting total tardiness of starts of to-follow surgeons in ORs) [15]. This is shown in Figure 1.

Previously, we developed Bayesian methods that can be used for accurate estimation of lower prediction bounds for OR times [11,16]. Conceptually, the method would apply equally well for surgical times. However, these Bayesian methods rely on having a prior distribution centered on the surgeon's and scheduler's estimates [11,16]. At the studied hospital, cases were scheduled based on historical OR times, not surgical times [11,16]. Therefore, there was no prior distribution for the surgical times. This absence precluded use of such Bayesian methods [11,16]. We thus relied on the original, older, technique developed for lower prediction bounds, that being to use the frequentist model based on the two-parameter lognormal distribution. For each procedure, historical data provided a sample size, sample mean of logarithms of surgical times, and sample standard deviation of the logarithms of surgical times. The 5.0% lower prediction bound was calculated in the log-scale using the Student t-distribution. Then, the exponential was taken of the result [14]. That is how Figure 1 was created from the data of Figure 2. Another alternative would have been to use nonparametric prediction bounds [14]. However, the parametric method’s estimates have greater precision than those estimated using nonparametric methods [17]. In addition, there must be at least 19 historical data for a 5% lower nonparametric prediction bound, but as few as two historical data are sufficient for the Student t-distribution [14,17].

There can be changes over time in surgical times. Therefore, as done previously, when calculating prediction bounds, for procedures with more than 99 previous cases, we limited use to the most recent 99 cases [18,19].

Prior data suggested the applicability of the two-parameter lognormal model to surgical times. Strum et al. compared the lognormal and the normal distribution for modeling surgical times, also defined as incision to closure complete [20]. Using the Shapiro-Wilk test, and a P < 0.01 criterion for the rejection of the lognormal or normal, among 1,580 combinations of anesthesia and Current Procedural Terminology codes, there were 40 rejected by both, 269 better fitting lognormal than normal, 66 better fitting normal than lognormal, and 1205 rejected by neither, principally due to small sample sizes. The ratio of 269 to 66 is an odds ratio of 4.08 for lognormal distributions superior to normal distributions, exact 99% confidence interval 2.87 to 5.93. More importantly, from Strum et al.’s Table 5, the normal model vastly underestimated the left tail (i.e., the segment of concern in our current paper) [20]. For a common procedure with 241 historical cases and a 10th percentile of 59 minutes for the surgical time, the lognormal model’s estimated 51 minutes was closer to the 59 minutes than was the normal model’s estimate of 29 minutes [20].

Nominally, the 5.0% lower prediction bounds calculated from the three years of historical data should be underestimated by 5.0% of future cases [14]. However, the probability distributions of surgical times are not two-parameter lognormal for all procedures [20]. Furthermore, surgeons performing the procedures change over time, technology advancements are implemented, etc. Table 2 shows that the observed incidence achieved by our 5% lower prediction bounds was 4.86% (99% confidence interval 4.55% to 5.19%). Table 2 also shows that the limitation that there had to be at least two historical data for each new case excluded only 2.0% of cases in the contemporaneous period..

Details of calculations with the cases

From October 2019 through September 2020 there were 30,987 cases performed at one of three surgical suites (Table 1). Each of those cases was matched by the primary surgical Healthcare Common Procedure Coding System code used for anesthesia billing to the 95,146 cases from the previous three years at any anesthetizing location (Table 1). For 99.5% of cases comprising the historical period, the procedure code was a surgical Current Procedural Terminology code. Among the 2596 procedures comprising the contemporaneous period, the 3479 procedures from the historical period provided 0 historical cases for 8% (209/2596) of procedures and three or fewer historical cases (i.e., as shown in Figure 1) for 26% (673/2596) of the procedures. Among the 30,987 cases during the year being studied, there were at least two historical cases of the procedure for 30,357 cases (i.e., 2.0% missing) (Table 2). The 5.0% lower prediction bound for the case during the one‑year contemporaneous period was taken as the value from the three years of historical data.

We calculated the available periods for breast milk pumping sessions for the “Cases reliably with ≥ 30 min available for a breast pumping session” rows in Table 3. For the starting time, the first available minute was 15 minutes after the start of surgery. The end of the available time was considered the time of the start of surgery plus the 5% lower prediction bound, unless the case finished sooner, in which event it was when the case finished. When the 5% lower prediction bound was less than 15 minutes, the case had no suitable period. Table 3 gives the percentages of cases with 5% lower prediction bounds for which the difference of the ending available time from the starting available time was at least 30 minutes.

**Table 3:** Periods with at Least 30 Minutes from 15 Minutes After the Start of Surgery Until the End of the Surgery Abbreviations: CL, confidence limit; min, minute ^a^ Measured as the interval from 15 minutes after the start of the surgery until the end of surgery. ^b^ The percentages in the 3rd row are reported without decimal places; the unrounded values can be calculated by dividing the corresponding numerator and denominators in rows 2 and 1, respectively. The 99% two-sided confidence intervals are Clopper-Pearson.

	All Cases Combined	Ambulatory Surgery Center	Children's Hospital	Children's Hospital Elective Cases	Adult Surgical Suite	Adult Surgical Suite Elective Cases
Total cases	30,357	7987	4872	4163	17,498	13,411
Cases reliably with $\geq$ 30 min available for a breast pumping session^a^	11,932	1439	1009	882	9484	8012
%, of cases with a reliable 30-min interval^b^	39%	18%	21%	21%	54%	60%
Lower 99% CL	39%	17%	19%	20%	53%	59%
Upper 99% CL	40%	19%	22%	23%	55%	61%

Statistical methods to interpret results

Table 2 and Table 3 have results listed as percentages of cases with calculated 5% lower prediction bounds. The exact 99% two-sided confidence intervals for the percentages were calculated using the conservative Clopper-Pearson method. The P-values are exact, calculated using the binomial method. Figure 2 was created using STATA 16.1 (StataCorp, College Station, TX). The rest of the work was done using Excel, Office 365 Version 2002 (Microsoft, Redmond, WA).

## Results

Approximately 39% of cases (99% confidence interval 39% to 40%, P < 0.0001) were reliably long enough for a 30-minute breast milk pumping session for the anesthesia provider delivering care in the OR, beginning no sooner than 15 minutes after surgery start, and ending before dressing was on the patient (Table 3). Our hypothesis that, overall, most (> 50%) cases would not be suitable for such sessions was confirmed. The percentages were heterogeneous among surgical suites, being 18% (17% to 19%) at the ambulatory surgery center versus 60% (59% to 61%) at the inpatient surgical suite among elective cases (Table 3).

These percentages of 18% and 60% (Table 3) were substantially less than the corresponding percentages of surgical times being at least 45 minutes, 55.8% and 85.7% (Table 1), with ratios of 1.43 and 3.10, respectively. That was because the lower 5% prediction bounds accurately (Table 2) accounted for the uncertainty in the duration of each case (Figure 1). Another way to draw the distinction was for the most common neurosurgical procedures, many of which were not quite common. Table 4 lists the median, 5th percentiles, and 5% lower prediction bounds for the most common of the neurosurgical procedures performed at the hospital studied.

**Table 4:** Surgical Times of the 50 most Common Neurosurgical Procedures at the Studied Hospital in 2019 Abbreviations: CPT^©^ = Current Procedural Terminology code, IQR = interquartile range ^a^  Among the 2936 Neurosurgery department cases during the contemporaneous 1-year period, there were 248 different HCPCS codes, each corresponding to a CPT code. There were 5 cases in which the surgical code was missing. Among those 248 different HCPCS, there were 18 with at least 50 cases; those are listed. ^b^  Note that there are a few 5.0% lower prediction bounds greater than the observed 5th percentile. That is because the percentiles are from the subsequent 1 year. In addition, the 5th percentile is a point estimate based on the sample size and the prediction bound is a calculated quantity.

CPT code	Cases^a^	5.0% Lower Prediction Bound	Surgical Time, 5th Percentile^b^	Median Surgical Time (IQR)	Description
61210	143	9	12	27 (19, 43)	Burr hole(s); for implanting ventricular catheter, reservoir, EEG electrode(s), pressure recording device, or other cerebral monitoring device (separate procedure)
36226	51	11	12	27 (18, 54)	Selective catheter placement, vertebral artery, unilateral, with angiography of the ipsilateral vertebral circulation and all associated radiological supervision and interpretation, includes angiography of the cervicocerebral arch, when performed
36224	84	14	11	28 (18, 41)	Selective catheter placement, internal carotid artery, unilateral, with angiography of the ipsilateral intracranial carotid circulation and all associated radiological supervision and interpretation, includes angiography of the extracranial carotid and cervicocerebral arch, when performed
20205	50	14	20	30 (25, 40)	Biopsy, muscle; deep
61154	89	19	21	36 (28, 48)	Burr hole(s) with evacuation and/or drainage of hematoma, extradural or subdural
61624	80	22	27	61 (44, 112)	Transcatheter permanent occlusion or embolization (e.g., for tumor destruction, to achieve hemostasis, to occlude a vascular malformation), percutaneous, any method; central nervous system (intracranial, spinal cord)
61886	120	23	15	37 (21, 47)	Insertion or replacement of cranial neurostimulator pulse generator or receiver, direct or inductive coupling; with connection to 2 or more electrode arrays
62230	51	33	32	67 (51, 90)	Replacement or revision of cerebrospinal fluid shunt, obstructed valve, or distal catheter in shunt system
62223	105	37	38	62 (50, 76)	Creation of shunt; ventriculo-peritoneal, -pleural, other terminus
63030	83	46	63	111 (93, 135)	Laminotomy (hemilaminectomy), with decompression of nerve root(s), including partial facetectomy, foraminotomy and/or excision of herniated intervertebral disc; 1 interspace, lumbar
61312	53	47	39	85 (60, 112)	Craniectomy or craniotomy for evacuation of hematoma, supratentorial; extradural or subdural
63650	54	50	50	70 (61, 85)	Percutaneous implantation of neurostimulator electrode array, epidural
61322	73	55	45	81 (65, 102)	Craniectomy or craniotomy, decompressive, with or without duraplasty, for treatment of intracranial hypertension, without evacuation of associated intraparenchymal hematoma; without lobectomy
61510	155	63	90	190 (136, 242)	Craniectomy, trephination, bone flap craniotomy; for excision of brain tumor, supratentorial, except meningioma
22551	86	66	97	158 (127, 212)	Arthrodesis, anterior interbody, including disc space preparation, discectomy, osteophytectomy and decompression of spinal cord and/or nerve roots; cervical below C2
22633	96	81	159	259 (210, 312)	Arthrodesis, combined posterior or posterolateral technique with posterior interbody technique including laminectomy and/or discectomy sufficient to prepare interspace (other than for decompression), single interspace and segment; lumbar
22840	62	102	111	183 (142, 230)	Posterior non-segmental instrumentation (e.g., Harrington rod technique, pedicle fixation across 1 interspace, atlantoaxial transarticular screw fixation, sublaminar wiring at C1, facet screw fixation) (List separately in addition to code for primary procedure)
22842	108	107	126	235 (185, 310)	Posterior segmental instrumentation (e.g., pedicle fixation, dual rods with multiple hooks and sublaminar wires); 3 to 6 vertebral segments (List separately in addition to code for primary procedure)

## Discussion

Implications of results for daily assignments of anesthesia residents or nurse anesthetists

For organizations to arrange for anesthesia residents or nurse anesthetists in ORs to be relieved from cases for breast milk pumping sessions, enough anesthesia providers must be available to give breaks. That was known before our study and, of course, is uninfluenced by the results other than to the extent that they reinforce that need. In the USA, providing adequate time for lactating women to express their breast milk is required by law, and, for resident training programs, it is required by the ACGME [2,3]. Alternatively, there could be a prolonged turnover between cases for the breast milk pumping session [21]. For surgical suites with > eight-hour workdays like the studied hospital, deliberately prolonging turnovers would be irrational (costs vastly greater than an extra anesthesia provider for breaks) and inconsistent with (in this context, appropriate) biases [22]. Before our study, it was known also, qualitatively, that the choice of anesthesia assignments can facilitate the approximately 30 minutes needed for breast milk pumping sessions [12]. For example, it was clear that providing such relief would be practical at a large, inpatient surgical suite with many long (e.g., seven-hour) cases but not at a freestanding gastroenterology clinic with many short duration (e.g., 30-minute) cases. The value of our findings is the quantification of the challenges involved in reliably providing time for these sessions. There are vastly more (Table 1 versus Table 3, ratios of 1.43 and 3.10) cases that may appear to be long enough for a breast milk pumping session by naively considering the percentage of surgical times at least 45 minutes long, rather than the appropriate question of how many are reliably (i.e., with ≥ 95% confidence) of such duration. This mathematical result will not be changed by other conceptual goals, just as they will not change the biology of breast milk and breastfeeding and the associated regulations [1-3]. The implication of our results for anesthesia groups and anesthesia providers who are breastfeeding their children is that not only does there need to be the providers to give the breaks but that recipients need to be assigned to ORs with one or more long-duration cases, a practice that may involve adjustments to their preferred clinical assignments and/or sequence of training rotations. For example, when an infant is younger, breaks for breast milk pumping sessions are needed more frequently. Thus, a non-OR rotation would have advantages compared to a pediatric ambulatory surgery center rotation. The value of our mathematical study is that consideration of the averages or even the 5th percentiles of historical surgical times gives a highly false impression that breast milk pumping sessions should have been possible. The impression is false because surgical times have substantial variability.

Other limitations

Our study was from one hospital. We can, however, make some comparisons with other organizations that relate to the generalizability of the findings. First, Bravo et al. found, for three years of cases at Boston Children’s hospital, that there were 17% of procedures with 30 or more observations encompassing 78% of cases [23]. We had 18% of procedures encompassing 68% of cases (i.e., similar). Second, Strum et al. and Bravo et al. found that relative variability was greater for shorter than for longer procedures [20,23]. Likewise, for the adult surgical suite elective cases with the greatest percentage of cases with surgical time at least 45 minutes, the ratio of that 85.7% from Table 1 to the percentage of cases with the 5% lower prediction bound at least 45 minutes, 60% from Table 3, equaled 1.4. In comparison, the ratio for the ambulatory surgery center with the briefest cases was 55.8% to 18% equaling 3.1, thus matching.

We performed our analyses by surgical suite, not by surgical specialty per se because many procedures were uncommon. There is substantial diversity in the types of procedures performed at the hospital studied. There also is small similarity in the probability distributions of procedures between cases with versus without breaks (0.25 [SE 0.01] on 0 to 1 scale). The latter result is simply because the incidences of cases sufficiently long for a break differ among procedures (e.g., no opportunity during extracapsular cataract removal). The consequence was that the sample sizes sufficient to consider breaks could not be achieved while controlling for the procedure itself [24]. Had we done so, the prediction bound would be even smaller and, therefore, our conclusions even more reliable.

We chose not to consider the potential impact of breaks on patient outcomes. First, brief breaks without permanent handoff are common. At the study hospital, the anesthesia providers received breaks for 41.9% of cases [24]. Second, at another large teaching hospital, the percentage was 37.6%. There, breaks were associated with a reduction (not increase) in the adjusted odds of a composite outcome of in-hospital mortality and major morbidities (0.92, 95% confidence interval 0.88 to 0.98, P < 0.0001) [25].

Finally, we did not consider the situation where supervising anesthesiologists give the breaks for breast milk pumping sessions. When the anesthesiologist is supervising two anesthesia residents, they are subject to regulations and billing rules, notwithstanding patient care issues related to immediate availability. When supervising three or four nurse anesthetists, there is a low chance of having suitable periods of multiple cases reliably aligning and not to have unexpected events in any of those ORs [26]. Our approach and issues raised would be moot when, toward the end of the workday, the anesthesiologist is supervising only one OR.

## Conclusions

We found that the 5% lower prediction bounds of surgical times can be estimated accurately based on two-parameter lognormal distributions for times classified by primary surgical procedure. We then applied the mathematics to quantify the percentages of surgical times that were reliably (≥ 95% probability) lasting at least 45 minutes, providing 30-minutes for breast milk pumping sessions without potentially interfering with patient care. The implications are that most surgical cases are not reliably of sufficient duration to accommodate relief of anesthesia providers for a 30-minute session. Individuals making OR assignments for anesthesia providers need to consider the 5% lower prediction bounds of such available time for cases in the room when making such assignments for women who require time for a breast milk pumping session. We showed that not only is it necessary to consider the lower prediction bounds, but also showed how to calculate them (e.g., using a spreadsheet program or equivalent). Alternative or complementary strategies involve changes in preferred assignments for the anesthesia providers and alteration in the order of rotations for anesthesia residents.
